# The dysregulation of immune cells induced by uric acid: mechanisms of inflammation associated with hyperuricemia and its complications

**DOI:** 10.3389/fimmu.2023.1282890

**Published:** 2023-11-20

**Authors:** Delun Li, Siyu Yuan, Yiyao Deng, Xiaowan Wang, Shouhai Wu, Xuesheng Chen, Yimeng Li, Jianting Ouyang, Danyao Lin, Haohao Quan, Xinwen Fu, Chuang Li, Wei Mao

**Affiliations:** ^1^ State Key Laboratory of Dampness Syndrome of Chinese Medicine, The Second Affiliated Hospital of Guangzhou University of Chinese Medicine, Guangzhou, China; ^2^ The Second Clinical Medical College, Guangzhou University of Chinese Medicine, Guangzhou, China; ^3^ Department of Nephrology, The Second Affiliated Hospital of Guangzhou University of Chinese Medicine (Guangdong Provincial Hospital of Chinese Medicine), Guangzhou, China; ^4^ Nephrology Institute of Guangdong Provincial Academy of Chinese Medical Sciences (NIGH-CM), Guangzhou, China; ^5^ Ministry of Education Key Laboratory of Pharmacology of Traditional Chinese Medical Formulae, Tianjin University of Traditional Chinese Medicine, Tianjin, China; ^6^ School of Chinese Materia Medica, Tianjin University of Traditional Chinese Medicine, Tianjin, China; ^7^ State Key Laboratory of Component-based Chinese Medicine, Tianjin University of Traditional Chinese Medicine, Tianjin, China; ^8^ Department of Nephrology, Guizhou Provincial People’s Hospital, Guiyang, Guizhou, China

**Keywords:** uric acid (UA), immune cell, gouty arthritis, MSU, sUA, serum uric acid

## Abstract

Changes in lifestyle induce an increase in patients with hyperuricemia (HUA), leading to gout, gouty arthritis, renal damage, and cardiovascular injury. There is a strong inflammatory response in the process of HUA, while dysregulation of immune cells, including monocytes, macrophages, and T cells, plays a crucial role in the inflammatory response. Recent studies have indicated that urate has a direct impact on immune cell populations, changes in cytokine expression, modifications in chemotaxis and differentiation, and the provocation of immune cells by intrinsic cells to cause the aforementioned conditions. Here we conducted a detailed review of the relationship among uric acid, immune response, and inflammatory status in hyperuricemia and its complications, providing new therapeutic targets and strategies.

## Graphical Abstract

In the hyperuricemic environment, sUA and MSU have direct or indirect effects on various immune cells. They can stimulate monocytes, macrophages, T cells, granulocytes, dendritic cells (DC cells), and innate lymphocytes.

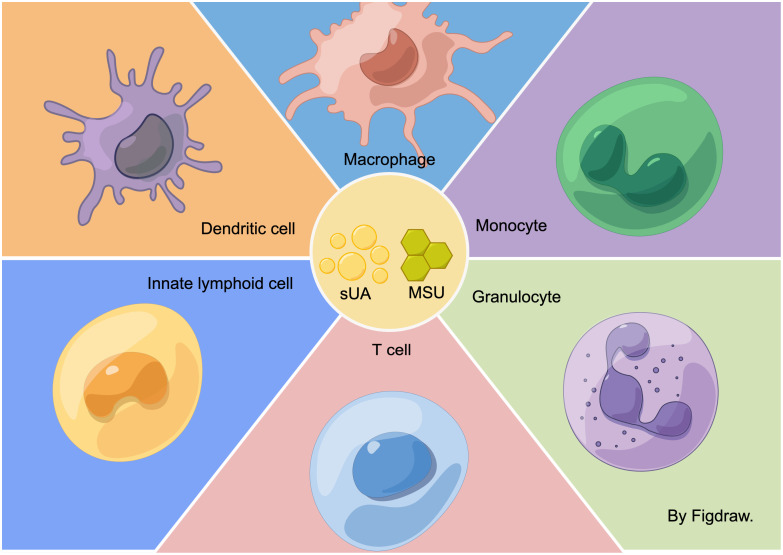

## Introduction

Uric Acid (UA) is the ultimate product of purine synthesis, occurring predominantly in the hepatic, intestinal, renal and vascular endothelial cells. This synthesis arises from dietary purines or the decomposition of endogenous purines, such as nucleic acids, adenine, and guanine derived from damaged and diseased cells (1). The kidney commands a leading function in UA excretion. Roughly 70% of the daily UA produced in humans is expelled by the kidneys (2), with the remaining 30% being eliminated *via* the intestines (3). Hyperuricemia (HUA) occurs when the production of UA exceeds its excretion, defined by a serum UA concentration of over 7.0 mg/DL (4). Traditionally, HUA was considered to solely cause gout and gouty arthritis. However, contemporary research has increasingly highlighted its impact on additional conditions, including renal disease, cardiovascular disease, hypertension, and achilles tendon rupture (5–9). Unraveling the regulatory mechanisms initiated by HUA is vital for understanding the development, progression, and treatment of its associated disorders.

Emerging research has delineated that HUA transcends the definition of a mere metabolic disease, extending to inflammatory and immune disorders. Intriguingly, HUA and its repercussions manifest within the circulatory system and the actual lesion site. This pervasive presence can be traced back to the infiltration of immune cells and an escalated overexpression of inflammatory cytokines by these immune cells (5, 9, 10). Uric acid has two states in the body: soluble urate (sUA) and monosodium urate (MSU) crystals. The progression of HUA and its complications is orchestrated through interactions among sUA, MSU crystals, or a mix of both coupled with innate immunity (5). Both sUA and MSU can perform as damage-associated molecular patterns (DAMP) to activate the natural immune response (11). Activation of natural immunity involves innate immune cells, such as macrophages, monocytes, NK cells and neutrophils, and their secretion of pro-inflammatory cytokines or induction of NACHT, LRR and PYD structural domain protein 3 (NLRP3) inflammasome activation, leading to a series of amplified inflammatory responses (12, 13). On the other hand, adaptive immunity can be induced either through the initiation of innate immunity or direct influence on T cells (14). Various studies denote that sUA or MSU crystals manifest a regulatory propensity on T cells, entailing their proliferation (15), recruitment (16), and polarization (17), Importantly, immune cells, under the regulation of UA in a modified state, have been identified as influential determinants guiding the advancement of HUA (16, 18). Despite the progress made in comprehending the mechanisms of UA-induced inflammation, there remains an ambiguity concerning how UA regulates immune cells involved in the inflammatory response in HUA and its complications. This ambiguity spans the operational changes in immune cells, alterations in cellular signaling pathways, and the interplay of pro-inflammatory responses between UA-triggered immune cells and tissue-intrinsic cells. The exact role of UA remains an enigma. It is crucial to elucidate the transformations of immune cells pertaining to HUA and its complications to facilitate a deeper exploration into the disease progression and therapeutic strategies.

In this review, our analysis concentrates primarily on the UA-induced activation of immune cells, the consequent cytokine expression, and detrimental inflammatory responses arising from the imbalance in the immune cell populations that contribute to the progression of HUA and its comorbidities. We summarize the effects of UA on immune cell-associated inflammatory responses from recent studies and debate the crucial role of immune cells in HUA and its associated disorders. Moreover, we propose novel clinical markers for screening therapeutic targets and approaches.

## UA’s direct impacts on immune cells

Differences exist in the regulatory effects of sUA and MSU on immune cells. More and more studies have shown that MSU activates natural immunity. It activates the expression of inflammatory signaling pathways through both direct contact with immune cell surface receptors and internalization by immune cells, inducing immune cell activation and promoting inflammatory responses (19). In contrast, the regulatory role of sUA on immune cells is controversial. Some studies have suggested that sUA was thought to be a preservation mechanism that evolved during human evolution due to its 90% reabsorption, implying that sUA has beneficial effects (20). Thus, sUA is thought to be a natural inhibitor of immune cells and could suppress inflammation induced by MSU (13, 21). Conversely, some studies have suggested that sUA can activate natural immunity in the same way as MSU and that sUA may also potentiate MSU activation of immune cells (22).

Although sUA and MSU have had opposite regulatory effects on immune cells in some studies, both have been known to impact on immune cells. In contrast, the role of MSU in activating immune cells may be more convincing. The other side of the immune-activating effect has recently been shown to require a specific concentration or to work in conjunction with other factors (21, 22). sUA’s regulatory effects on immune cells still need to be further investigated, therefore, the regulatory effects of sUA mentioned in the subsequent articles will be elaborated separately, so as to to demonstrate the diversity of sUA better, and to discuss the reasons affecting the regulatory effects of sUA.

## Monocytes

**Figure 1:**
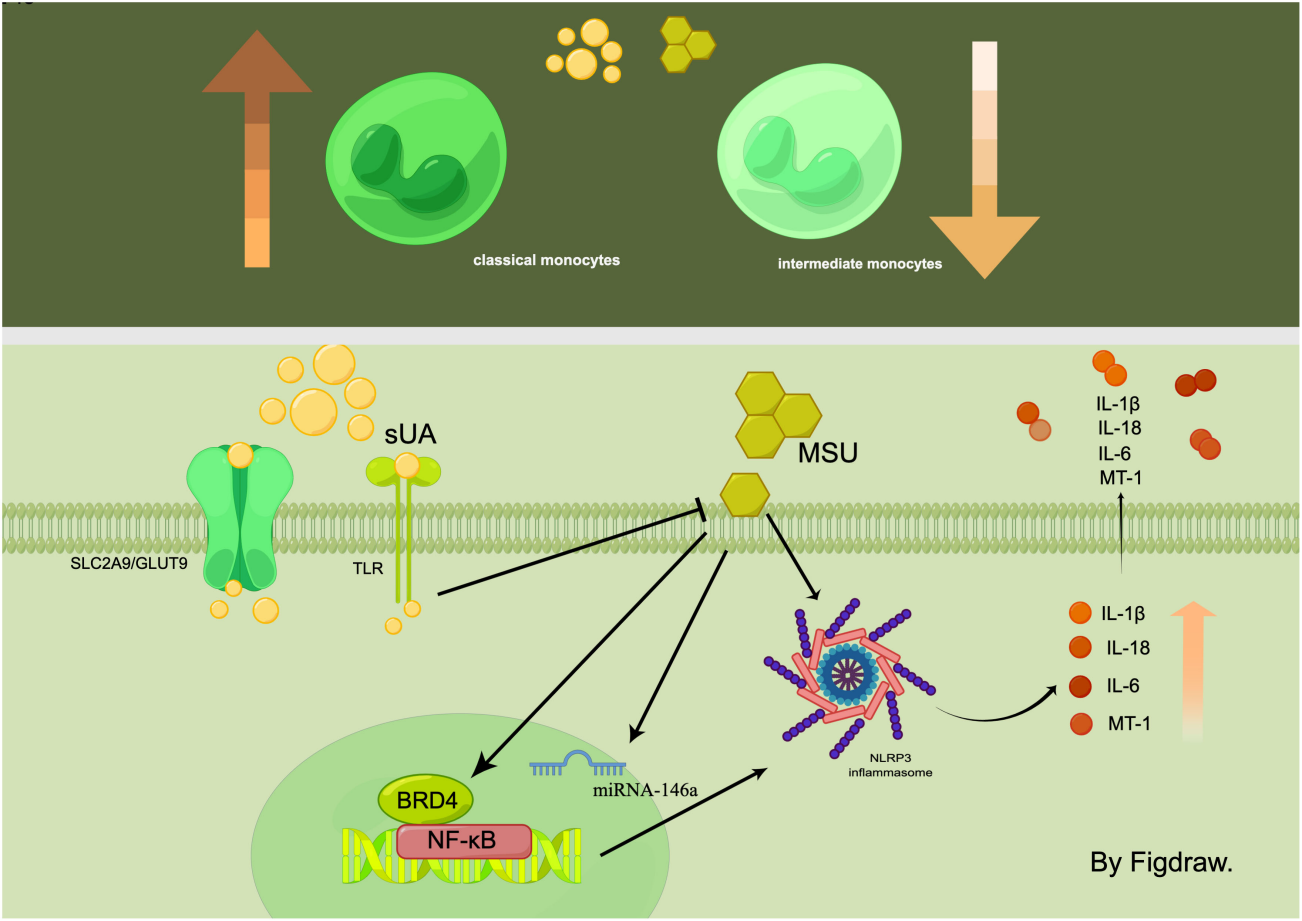
In the hyperuricemic environment, the number of classic monocytes increases while the number of intermediate monocytes decreases. sUA and MSU act on monocytes through multiple signalling pathways, such as NF-κB/NLRP3/GSDMD and SLC2A9/GLUT9, which ultimately affect the expression of inflammatory factors.

As progenitor cells for macrophages, monocytes, steer the UA-induced alterations in immune cells. Upon migration to peripheral tissues, monocytes differentiate into either dendritic cells or macrophages. They possess the unique adaptation to swiftly modify their functional phenotype in response to dynamic organismal environments, demonstrating considerable plasticity and heterogeneity (23). UA serves as an inducer of monocytes, with several studies indicating that UA transforms the epigenetic landscape of Peripheral Blood Mononuclear Cells (PBMC). Differential alterations, induced by UA, are evident in both histone modifications and DNA methylation, which suggests the potential of UA in altering the epigenetic inheritance of immune cells. Such alterations might contribute to the persistent inflammation in tissues, even after the dissolution of MSU crystals. Consequently, these epigenetic shifts observed in monocytes could serve as a novel target for gout therapeutics (24) (**Figure 1**).

## UA regulates monocyte populations and induces cytokine dysregulation

Differences were observed in the monocyte populations within the peripheral blood of gout patients compared to healthy individuals. Specifically, the plasma of gout patients exhibited enriched populations of classic monocytes, whereas intermediate monocytes were more prevalent in the plasma of healthy individuals (5). There was also a notable variation in the monocyte MicroRNA (miRNA) levels amongst these populations, with PBMC cells displaying significantly increased miRNA-146a levels in HUA-critical gout patients. Interestingly, a significant decrease was observed in the expression of miRNA-146a in PBMCs derived from gout patients. *In vitro* assays delineated MSU crystals’ propensity to induce TH1 cells to overexpress miRNA-146a, implying that miRNA-146a acted as a transcriptional brake in monocytes, which was suppressed during acute inflammatory responses to MSU crystals. Metallothionein-1 (MT-1) mRNA levels were markedly elevated during exacerbations in gout patients, particularly in PBMCs from patients with gouty stones. However, no significant difference in serum MT-1 levels was observed among inactive gout patients, healthy individuals, and those with HUA without gout. Additionally, a positive correlation was evident between serum MT-1 levels and C-reactive protein, as well as IL-1β, IL-6, and IL-18. Therefore, the differential expression and levels of monocyte populations, proteins, and miRNA suggest their potential utility as diagnostic, predictive, and differential markers for HUA and its related complications.

## sUA and MSU crystals regulate monocyte

MSU crystals induce monocyte-associated inflammation.MSU crystals directly instigate the assembly of NLRP3 inflammasome in monocytes, leading to an increase in IL-1β production (25, 26). Additionally, MSU induces monocyte cell pyroptosis *via* nuclear factor kappa-B(NF-κB)/NLRP3/gasdermin D(GSDMD) signaling pathway activation. This action occurs through its interaction with the Bromodomain Protein 4 (BRD4) in human monocytes, which subsequently contributes to IL-1β secretion and prompts an inflammatory response (27). sUA was also considered to exert a pro-inflammatory effect on monocytes. However, it’s imperative to note that its crystallization into MSU is required to activate the NLRP3 inflammasome in monocytes. Interestingly, sUA seems to augment the sensitivity of monocytes to MSU crystals (22). In addition to its pro-inflammatory effects, sUA also manifests as an immunosuppressive agent in monocytes (28). A study by QiuYue et al. discovered that monocytes absorb sUA intracellularly *via* the urate transporter protein solute carrier family 2, member 9 (SLC2A9)/glucose transporter 9 (GLUT9). This intracellular presence of sUA curbs monocytes’ ability to respond to inflammatory stimuli and inhibits their activation induced by MSU crystals. *In vitro* experiments further disclosed the inhibitory effect of sUA on the monocyte Toll-like receptor (TLR) signaling pathway, which in turn reduces the migration capability of classical monocytes (6). These findings support that sUA is more than just a substrate for MSU crystal formation. It also acts as an inherent inhibitor of tissue inflammation instigated by MSU crystal-induced monocyte activation (21).

## Macrophages

**Figure 2:**
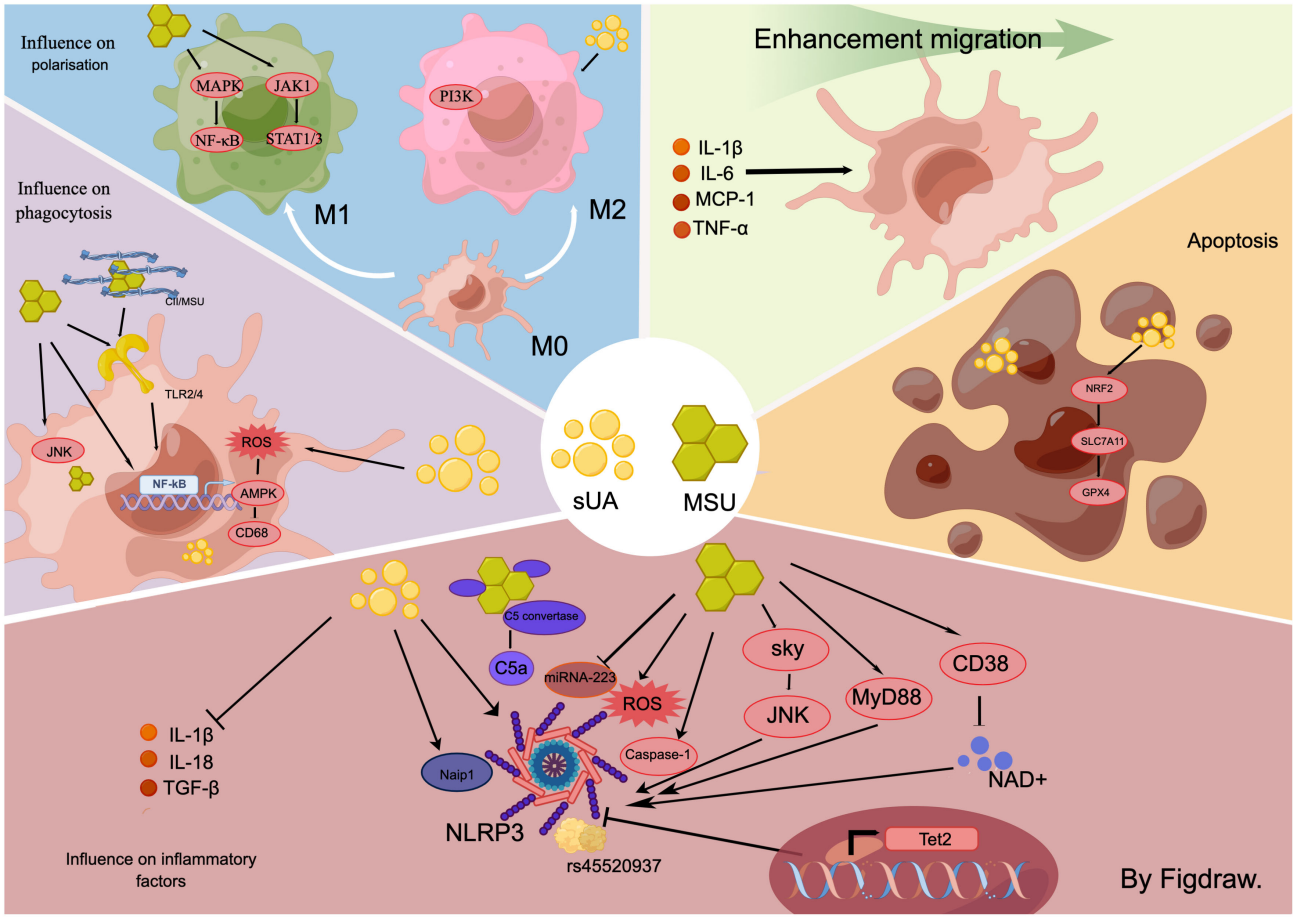
In the hyperuricemic environment, for macrophages, the effects of sUA and MSU are inconsistent. In general, both substances induce macrophage-associated inflammation through multiple signalling pathways, including sky/JNK and myD88/NLRP3. They also influence macrophage migration, disrupt the balance between M1 and M2 populations, activate macrophage phagocytosis, and promote the production of necrotic macrophages.

Macrophages in most tissues have diverse functions, including initiating immune and inflammatory responses toward pathogens, maintaining tissue homeostasis, and contributing to tissue repair and remodeling (29). A segment of the acute inflammatory response incited by HUA is attributable to macrophages’ phagocytosis of MSU crystals (25). Existing research poined towards significant macrophage infiltration in the tissues affected by HUA and its complications (5). Furthermore, a consensus links macrophages to metabolic diseases such as HUA (30), with metabolic reprogramming identified as a characteristic trait of macrophage activation (31). Recent findings from transcriptomic and metabolomic studies underscored the association between macrophage metabolic reprogramming and its functional malleability (32). Numerous factors present within the cellular microenvironment can be stimulated to modulate cellular metabolism, which can foster macrophage polarization and alter integral signaling pathways involved in that polarization. Significantly, macrophages play a crucial role in the pathogenesis of the inflammatory and autoimmune disorders seen in HUA and its subsequent complications (33, 34) (**Figure 2**).

## UA induces macrophages to produce inflammatory cytokines

MSU crystals induce macrophage production of inflammatory cytokines (35). Specifically, MSU directly initiates macrophage protein hydrolysis mediated by caspase-1, which processes the NLRP3 inflammasome and heightens the secretion of IL-1β and IL-18 (35). This mechanism forms a well-recognized signaling pathway for MSU-induced inflammatory responses in macrophages. Furthermore, MSU crystals can directly activate both dormant and active macrophages *via* the Syk and JNK kinase signaling pathways (36), stimulating the production of cytokines such as IL-1β, Transforming Growth Factor-beta (TGF-β), and others. These cytokines induce inflammation and metabolic reprogramming through either the MyD88-dependent pathway or the Reactive Oxygen Species (ROS)-NLRP3 signaling pathway (37–39). Thus, macrophage NLRP3 inflammasomes play a crucial role in MSU-induced macrophage-associated inflammation. The degeneration of NLRP3 directly mitigates the inflammatory response. Enhancing NLRP3 degeneration by amplifying macrophage autophagy stands as a powerful approach to counter macrophage inflammation (40). Additionally, MSU crystals diminish intracellular NAD+ concentration by stimulating the nicotinamide adenine dinucleotide (NAD+) degrading enzyme (CD38) in macrophages, subsequently promoting IL-1β secretion (41). Tet2 influences the macrophage’s response to the stimulation from monosodium urate (MSU) crystals. It was observed that *Tet2* knockout mice exhibited an increase in IL-1ß secretion following MSU crystal administration. Additionally, macrophages depleted of *Tet2* secreted more IL-1β upon stimulation with MSU crystals, a phenomenon mitigated by the inhibition of NLRP3 inflammasomes. These findings suggest that *Tet2* is a transcriptional gene associated with macrophage NLRP3 inflammasomes and promoting its overexpression in macrophages could serve as a potential therapeutic approach to mitigate the inflammatory response in HUA (34). Recent studies have underscored the role of miRNAs in the down-regulation of inflammation in autoimmune diseases and inflammatory disorders (42). A comparative study of serum samples from acute gout (AG) patients, intergout gout (IG) patients, and healthy individuals acknowledged that the expression of miR-223 was significantly diminished in the AG group. However, the expression elevated post-acute gout remission. Subsequent investigations indicated that MSU crystals targeted the NLRP3 inflammasome by suppressing macrophage miR-223 expression and facilitated the production of inflammatory cytokines, IL-1ß and TNF-α (43). Moreover, MSU crystals also stimulated the secretion of macrophage inflammatory factors through the induction of genetic variants. Research has unveiled variants of the peroxisome PPARGC1B in macrophages derived from patients suffering from gouty arthritis, notably the missense single nucleotide polymorphism rs45520937. *In vitro* testing confirmed that MSU crystals trigger the manifestation of macrophage rs45520937 and amplify the expression of NLRP3 and IL-1β (44). In HUA and its ensuing complications, MSU crystals also escalate their role in prompting macrophages to produce inflammatory factors by mediating other cytokines such as leptin (45). The complement system serves as a nexus between innate and adaptive immunity (46). Its activation bolsters macrophage activation induced by MSU crystals. Functional C5 convertase complexes aggregate on the MSU crystal surfaces, culminating in the production of active C5a. C5a activates the NLRP3 inflammasome in macrophages and fosters the release of IL-1β (47), thereby contributing to the inflammation response triggered by HUA.

Studies pertaining to sUA-triggered innate immunity have shown differential results. (1) sUA activates innate immunity. Acts as a macrophage DAMP molecule like MSU crystals to promote hyperuricemia and its complications (47, 48). sUA incites the production of IL-1β *via* NLRP3 inflammasomes in macrophages (48), which in turn, stimulates tissue-intrinsic cells to induce NF-κB signaling pathway expression, thereby exacerbating the inflammatory response. (2) sUA inhibits macrophage. sUA serves as a natural immunosuppressant, which diminishes IL-1β production in mouse macrophages (28). During investigations into HUA complications, MSU crystals were observed to induce fibrosis and expedite the progression of hyperuricemic nephropathy, unlike sUA (17). Recent research indicates that Naip1 in mouse macrophages directly recognizes sUA and its expression in human macrophages induces IL-1β, a process reversible by pharmaceutically and genetically inhibiting NLRP3 (49). Additionally, Naip1-NLRP33 interaction was experimentally indicated, with the loss of Naip in human macrophages relieving IL-1β production post-sUA stimulation. The differential experimental outcomes are potentially attributable to variations in sUA concentrations and origins of the cell species.

## UA activates phagocytosis by macrophages

MSU crystals promote phagocytosis by macrophages. Phagocytosis by MSU crystals for foreign body elimination (50, 51). Despite their reduction through macrophage phagocytosis, the inflammatory response persists due to the continued macrophage engagement with MSU crystals (52), contributing to the chronic inflammation characteristic of HUA. This cyclical response is due to the activation of macrophage TLR 2 and 4, NF-κB, and JNK/Erk signaling pathways during phagocytosis, causing the secretion of pro-inflammatory cytokines IL-1β, IL-6, TNF-α, and Prostaglandin E2(PGE2) (51, 53). Recent studies indicated a more potent phagocytosis-inducing effect on macrophages when MSU crystals interact with tissue proteins (19). MSU crystals in synovial fluid of gouty arthritis patients are found to be enriched with type II collagen (CII). CII influences the morphology of single MSU crystals and how they are arranged in the eutectic system, thereby magnifying phagocytosis and oxidative stress in macrophages. Further, CII enhances the expression of the MSU-induced chemokines CXC motif chemokine ligand 2 (CXCL2), Chemokine (C-C motif) ligand 2 (CCL2), and the pro-inflammatory cytokine IL-1β in macrophages. Overall, CII stimulates the integrin β1 (ITGB1)-dependent TLR2/4-NF-κB signaling pathway in macrophages, intensifying MSU-induced inflammation. Therefore, in the environment where MSU is present, promoting phagocytosis of MSU or MSU protein complexes by macrophages, thereby accelerating the metabolism of MSU from the site of injury is a potentially effective way to inhibit inflammation generation. Moreover, MSU relies on ITGB1 on the surface of macrophages to activate the inflammatory signaling pathway, which suggests that targeting and inhibiting integrins on the surface of macrophages to inhibit inflammation in MSU-induced inflammatory complications is one of the research directions for precision therapy. Overall, simultaneous promotion of macrophage phagocytosis of MSU crystals and inhibition of MSU crystal-induced generation of inflammation may reduce the inflammatory response more rapidly.Therefore, inhibiting MSU crystal phagocytosis or reducing the expression of inflammatory factors post-phagocytosis may present an efficient therapeutic approach during MSU production. This hypothesis was confirmed in a recent study that the inflammation inhibitor IL-37 boosted MSU phagocytosis by macrophages, lowered the transcription of pyroptosis-associated proteins, and diminished the release of inflammatory cytokines post-phagocytosis, thereby mitigating the inflammatory response in gout (54). IL-37 has been recognized recently as a significant member of the interleukin family associated with developing and treating gout (55). There is growing evidence that IL-37 inhibitors can suppress the inflammatory response in gout by inhibiting multiple inflammatory signaling pathways and modulating macrophages. However, this inhibitor is still a long way from clinical use, and there are no studies yet to validate the systemic adverse effects of the non-targeted use of this immunosuppressive agent. We look forward to seeing more in-depth studies of IL-37 inhibitors in the near future and to clinical translation.

Studies examining the phagocytosis of macrophages by sUA have demonstrated varied results. (1) Inhibition of macrophage phagocytosis by sUA.Research into HUA-induced atherosclerosis has suggested that macrophage overexpression of xanthine oxidoreductase (XOR) and heightened intracellular sUA concentrations activate macrophage phagocytosis. This activity may lead macrophages to engulf large amounts of fat, forming macrophage foam cells and subsequent atherosclerosis (7). (2) sUA promotes phagocytosis by macrophages (56). Exposure to sUA triggered the macrophage ROS-AMPK pathway, impaired CD68 expression, decreased macrophage phagocytosis, and inhibited macrophage foam cell formation. These divergent outcomes of sUA’s impact on macrophages within the same disease study could be attributed to varied sources of sUA. Both studies employed intracellular and extracellular sUA to stimulate macrophages, respectively. Further investigation is warranted to clarify the immunomodulatory effects of sUA on macrophages.

## UA induces macrophages migration to produce inflammation

MSU has been found to directly induce migration in macrophages. Part of this migratory effect can be attributed to the stimulation of IL-1β secretion by resident macrophages *via* MSU crystals, which subsequently instigates caspase-11 expression in macrophages through IL-1R and MYD88 (55). The migration of circulating macrophages to MSU sites is induced by increased caspase-11 secretion. Moreover, macrophages possess the ability to relocate post-phagocytosis of encapsulated MSU crystals. Macrophages transporting these migrated MSU crystals may potentially contribute to the tissue’s widespread inflammatory response (57). Recent research also illustrates that curtailing macrophage migration to the MSU site effectively mitigates HUA’s inflammatory response and associated complications (58).

sUA triggers macrophage migration indirectly. HUA instigates insulin resistance (IR) in various peripheral tissues, a recognized complication of HUA (59). Initially, sUA activates resident liver macrophages, prompting them to produce pro-inflammatory cytokines, including IL-1β, IL-6, MCP-1, and TNF-α, thereby inducing the migration of circulating monocyte-derived macrophages toward the liver. Once reaching the liver, these macrophages are inhibited by sUA, which further prevents the nuclear translocation of GLUT-4 and impedes insulin receptor substrate 2 (IRS2)/Phosphoinositide 3-kinase (PI3K)/AKT signaling. Concurrently, sUA mediates the degradation pathway of the IRS2 protein and activates Adenosine 5’-monophosphate (AMP)-activated protein kinase (AMPK)/mammalian target of rapamycin (mTOR) in macrophages, thereby reducing energy consumption which leads to IR (60). Supplemental studies have shed light on the upstream mechanism of this signaling pathway, demonstrating that sUA provokes IR in HUA by stimulating the Nuclear factor erythroid 2-related factor 2 (Nrf2)/Heme Oxygenase 1(HO-1) signaling pathway and upregulating the regulatory thioredoxin interacting protein (TXNIP), thereby inhibiting the IRS2/PI3K/AKT signaling pathway in macrophages (57). In conclusion, sUA induces an inflammatory response at the site of injury by stimulating resident macrophages to recruit circulating immune cells (60).

## UA induces dysregulation of macrophage glucose metabolism to produce inflammation

Increased glucose uptake and glycolysis are metabolic markers of pro-inflammatory activation of immune cells. MSU activates macrophage glycolysis to promote inflammatory factor expression (58). Glycolysis regulates NLRP3 inflammasome activation in macrophages (61). High concentrations of glucose increase IL-1β production *via* an NLRP3-dependent pathway (62). MSU crystals increase glucose uptake in macrophages by upregulating glucose transporter protein 1 (GLUT1) and upregulating glycolysis. This triggers metabolic reprogramming, leading to NLRP3-dependent IL-1β production (58). Inhibition of both GLUT1 and glycolysis reduced MSU crystal-induced inflammation response. Notably, immune cells in tissues injured by HUA complications have higher GLUT1 expression than those in circulation (58). This suggests that targeting and inhibiting GLUT1 in macrophages is a promising and specific approach as a treatment for HUA and its complications.MSU crystals also induce inactivation of mitochondrial pyruvate carrier (MPC) in macrophages promoting NLRP3 inflammasome activation and gout development. Pioglitazone is a commonly used hypoglycemic agent in clinical practice, which also has an inhibitory effect on MPC. Therefore, the increased risk of NLRP3-associated autoinflammatory diseases should be considered in the clinical use of drugs such as pioglitazone that target MPC (61). In addition, *in vitro* assays in mouse macrophages revealed a significant increase in succinic acid expression upon glycolytic activation. Further experimental results suggest that succinate is a metabolite in innate immune signaling that enhances IL-1β production during inflammation by stabilizing hypoxia-inducible factor-1α (HIF-1α). Recent studies have also shown that the inflammatory response of macrophages can be attenuated by inhibiting succinic acid (63, 64). However, there is still a gap in the research on the modulation of macrophage inflammatory response through inhibition of succinate in HUA disease, so this aspect of the study is innovative for inhibiting HUA inflammatory response through the modulation of glycolysis.

sUA induces insulin resistance in macrophages. IR is one of the complications of HUA (59). sUA inhibits nuclear translocation of GLUT-4 in macrophages and blocks insulin IRS2/PI3K/AKT signaling. In addition, sUA mediates the IRS2 protein degradation pathway and activates AMPK/mTOR in macrophages, which reduces macrophage energy consumption leading to IR (60). Other studies have complemented the upstream of this signaling pathway and showed that sUA induces IR in HUA by activating the Nrf2/HO-1 signaling pathway through up-regulation of the regulatory thioredoxin-interacting protein (TXNIP) in macrophages that inhibits IRS2/PI3K/AKT signaling pathway (57). In conclusion, sUA impairs the macrophage glucose transport signaling pathway, thereby inhibiting glucose uptake and leading to IR (60). Although further *in vivo* studies are needed, TXNIP and Nrf2 inhibitors may be promising therapeutic targets for preventing and treating HUA-induced insulin resistance in macrophages.

## UA induces necrosis of macrophages to produce inflammation

Recent research suggests that UA-induced necrosis significantly contributes to HUA and its associated complications. MSU crystals can stimulate necroinflammatory responses; therefore, inhibiting this inflammation, induced by MSU crystals, may present a therapeutic approach to managing HUA and related complications (65). When exposed to MSU crystals, mouse macrophages underwent various forms of cell death, including pyroptosis, apoptosis, and necroptosis. However, the induction of necroptosis in mouse macrophages was not effectively prevented by NLRP3 inhibition (66), indicating that MSU crystals may stimulate macrophage necrosis and ensuing inflammation through various mechanisms. This discovery holds consistent when observing sUA, which also prompts macrophage necrosis, manifesting HUA complications. For instance, in an investigation of atherosclerosis induced by HUA, exposure to sUA significantly heightened macrophage iron death through the NRF2/SLC7A11/Glutathione peroxidase 4 (GPX4) signaling pathway (67). This reaction hampered autophagy, fostering the development of atherosclerosis in conjunction with HUA. A deeper understanding of the inflammatory pathways invoked by UA-induced macrophage necrosis might shed light on the constant state of inflammation characteristic of HUA and its associated complications.

## UA induces macrophage polarization to produce inflammation

M1/M2 populace dysregulation is evident in HUA and its associated complications (35, 68). Changes to the macrophage population could significantly affect UA concentrations, subsequently amplifying HUA complications. The recently identified metabolism-related gene, CG9005 (*mda*), plays a crucial role in the innate immune response within HUA. The gene showed low activity in stationary macrophages (M0), increases in pro-inflammatory differentiated cells (M1 macrophages), and decreases in cells differentiating into anti-inflammatory macrophages (M2 macrophages) (69). By inhibiting *mda*, the manifestations of HUA could be reduced, aligning with the examination of macrophage population dysregulation in HUA: the harmful capacity of M1 macrophages and the remedying potential of M2 macrophage therapy. Therefore, understanding the variations in macrophage polarization in HUA and related complications carries considerable implications for pathogenesis research and therapeutic approaches.

MSU crystals induced macrophage polarization towards the M1 type. MSU crystals activate the Mitogen-activated protein kinase (MAPK)/NF-κB and Janus kinase-1 (JAK-1) - signal transducers and activators of transcription1/3 (STAT1/3) signaling pathways in macrophages, inducing M1 macrophages polarization and releasing M1 macrophages-associated factors TNFα, IL-1β, and IL-6 (66, 70). It was found in a hyperuricemic kidney injury study found that modulating M1 macrophages dampened the inflammatory response (17). Other study determined that activation of AMPK/sirtuin 1 (SIRT1) stifled M1 macrophage polarization and alleviated the inflammatory response in HUA (71). It was also established that besides MSU crystals alone, macrophages engulfing MSU crystals and creating granulomas could further spur the generation of M1 macrophage polarization, intensifying interstitial inflammation and propagating chronic kidney disease (CKD) progression (72). Therefore, strategic reduction of M1 macrophages or their cytokine expression could potentially alleviate the inflammatory response in HUA and its associated complications.

sUA induces macrophage polarization toward the M2 type. The M2 macrophages are characterized as an anti-inflammatory phenotype, demonstrating high Arg-1 expression and producing anti-inflammatory effects (73, 74). Inducing M2 macrophage polarization in macrophages present at inflammation sites can effectively lessen the inflammatory response. sUA exhibits immunosuppressive properties, partially realized through the induction of macrophage polarization toward the M2 macrophages. Studies conducted on an asymptomatic mouse model of HUA, subjected to acute kidney injury instigated by ischemia-reperfusion (IR-AKI), revealed that sUA could drive macrophage polarization toward the M2 macrophages, thereby expediting the recovery of renal function and structure in IR-AKI (75). Furthermore, the activation of the PI3K/Akt signaling pathway in macrophages during gouty arthritis studies has been found to induce an anti-inflammatory effect by promoting a shift toward the M2 macrophages, ultimately mitigating MSU-induced gouty arthritis (76).

## Granulocytes

Granulocytes, innate immune cells comprising neutrophils, eosinophils, basophils, and mast cells, depend on inflammatory signals for their recruitment to sites of injury, infection, or allergic reactions. These signals, in turn, activate the granulocytes to release immunostimulatory molecules (77). Notably, research pertaining to HUA and its ensuing complications has predominantly focused on neutrophils.

## Direct effect of UA on neutrophils

MSU crystals induce neutrophil migration (47), playing a critical role in acute inflammatory responses, including infections and sterile injuries characterized by crystal deposition. During sterile inflammation, neutrophil recruitment from the bloodstream to inflamed tissues incorporates various pro-inflammatory chemokines and cytokines such as CXCL8, TNF-α, and IL-1β. The migration process involves distinct stages, including adhesion, rolling, and crawling, which significantly necessitate β2 integrins (78). MSU crystals directly enhance CD18 integrin activation in neutrophils (79), augmenting their ability to migrate toward these crystals *via* leptin-facilitated mechanisms (45). Following recruitment, these crystals latch onto the neutrophil plasma membrane lipids directly, instigating the lipid raft structural domains’ aggregation in the membrane, thereby activating the Syk kinase. This activation leads to increased neutrophil activity and cytokine production (6). Furthermore, MSU crystals can trigger Neutrophil Extracellular Traps (NETs), whose formation leads to the release of nuclear DNA bundled with neutrophil enzymes. NETs play a pivotal role in the commencement and progression of inflammation in gout, presenting the primary methodology through which neutrophils facilitate inflammation resolution (55, 80, 81).

sUA inhibits neutrophil migration. sUA undermines β2 integrin activation and signaling, consequently diminishing neutrophil migration to inflammation sites *in vivo*. Upon entry into neutrophils *via* the SLC2A9 urate transporter protein, sUA regulates the intracellular pH, modifies cytoskeletal dynamics to reduce cellular size, and manages β2 integrin activity as well as its internalization/recycling, leading to an overall decrease in neutrophil functions such as migration. In addition to these observations, sUA is also noted to curtail cytokine release and phagocytosis capabilities without influencing NETs release (17, 82). Interestingly, impeding the intracellular uptake of sUA by urate transporter proteins can reverse the inhibitory effect of sUA on neutrophils.

## UA’s indirect impact on neutrophils

Apart from direct stimulation of granulocytes, MSU crystals can also modulate the immune microenvironment indirectly by affecting other immune cells. UA indirectly triggers the inflow and activation of neutrophils to inflammation sites by spurring macrophages to produce the IL-1β, which tends to reach the inflammation site first (47). This stimulation of IL-1β production by macrophages significantly enhances neutrophil recruitment and intensifies the inflammatory response incited by HUA (83). Alongside IL-1β, MSU crystals also induce macrophages to produce caspase-11, a module that serves both as a recruiting agent for circulating neutrophils and a stimulatory cytokine prompting neutrophils to form NETs. In-depth studies manifested that caspase-11 orchestrates neutrophil chemotaxis and extracellular trap formation by advocating the phosphorylation of the neutrophil filament-cutting protein, Cofilin (55). An alternate pathway exists wherein resident macrophages initially clear small urate microaggregates (UMAs) formed due to HUA. However, if clearance fails, these UMAs undergo bipolar growth to forge standard full-sized needle-like monosodium urate crystals (nsMSUs) which directly induce neutrophils, resulting in the formation of NETs (84). Notably, the phagocytosis of neutrophils was also shown to be affected in the results of this study. Neutrophil phagocytosis is an essential function for host defense against infection. Moreover, impaired neutrophil phagocytosis can lead to the development of other diseases due to immunodeficiency. Therefore, further investigation of the role of neutrophils in disease development, where phagocytosis is affected by soluble uric acid, may be worthwhile in order to clarify whether sUA is indeed a beneficial immunosuppressive agent.

## Dendritic cells

DCs are intrinsic in the natural immune system where these recognize pathogens and activate immune cells in the adaptive immune system (85). The initiation of the adaptive immune mechanism by DCs is realized by inducing naïve T-cell activation and differentiation *via* major histocompatibility complex (MHC) molecules that present antigenic peptides. Sequentially, DCs persistently induce effector T cell differentiation and govern T cell tolerance (86). Moreover, DCs are capable of secreting cytokines and growth factors to reinforce and manage the immune response (87).

## Populations of DCs display imbalance in the complications associated with HUA

A higher count of DCs was observed in the plasma of healthy individuals compared to those suffering from AG (5). This observation suggests that MSU crystals could induce the migration of DCs, culminating in a diminished number of circulating DCs. The critical role of UA as a catalyst modulating the functionality of DCs has been verified; it facilitates DCs maturation, bolsters the presentation of foreign antigens by DCs, and prompts the stimulation of T lymphocytes (88).

## T cells

Elevated purine levels in HUA bolster the proliferative response of T lymphocytes towards pro-mitotic and antigenic stimuli (89). It was determined that uric acid could independently activate T cells, irrespective of the presence of antigens. Experimental findings with human T cells isolated from healthy human blood samples indicated that UA not only escalates T cells activation by CD25 expression, but also encourages IL-1β secretion in an NLRP3 inflammasome-dependent manner, while simultaneously promoting T cell proliferation (90).

Furthermore, UA intensifies the immune response of CD8^+^ T cells by provoking DCs and macrophages to express the co-stimulatory molecules CD80 and CD86 (88). Allopurinol, a prevalent treatment for symptomatic HUA or gout, has been demonstrated to mitigate T cell activation following CD3 and CD28 antigen stimulation. This is evident from the decreased CD69 expression and diminished secretion of IL-2, a cytokine influential in driving T cell activation, and IFN-γ, a critical effector of Th1. Such findings propose that directing interventions toward pro-inflammatory T cells and corresponding cytokines proves hopeful as a strategy in preventing and treating gout.

Deviances within T-cell populations have implied significance in HUA research, proposing that therapeutic interventions could be directed at these T cells. Dipeptidyl peptidase 4 (DPP4), also recognized as CD26, is manifested on macrophages and T helper cells (Th1, Th2, Th17). The expression of DPP4 may enhance in response to Th cells activation, and can co-stimulate T cells activation and proliferation (91, 92). DPP4 is also discernible in plasma, proximal tubules, peduncles, and the brush border of glomerular endothelial cells (93, 94). Hence, disrupting the influence of DPP4 on T cells potentially represents a modality for treating systemic complications of HUA manifesting in renal tissues.

**Figure 3:**
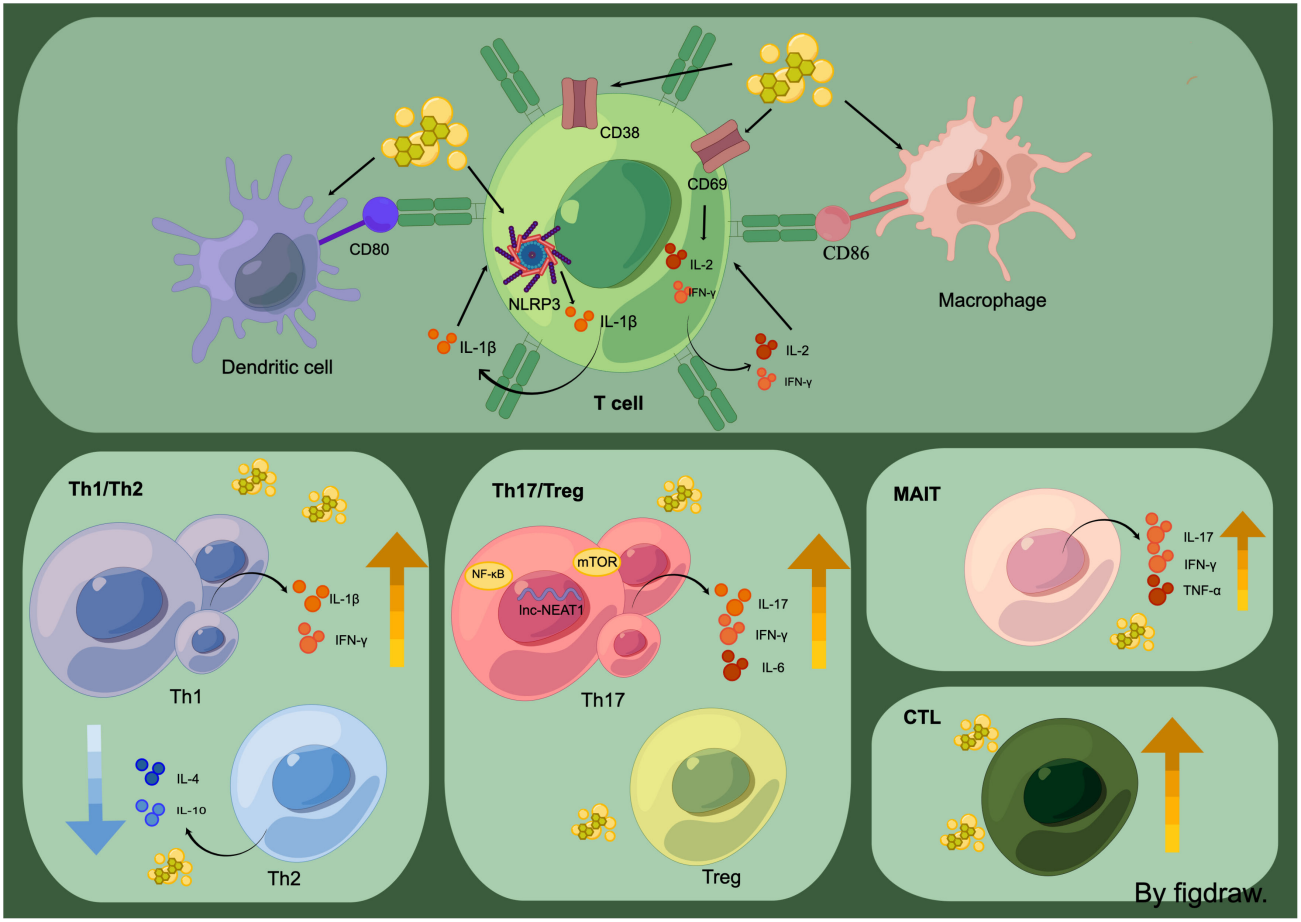
T cells, in the high-uric acid environment, show increased expression of inflammatory factors due to the direct stimulation of T-cell receptors like CD38 by sUA and MSU. Additionally, DC cells and macrophages can activate T cells through CD80 and CD86 receptors. The high-uric acid environment also leads to dysregulation of T-cell populations, including Th1/Th2, Th17/Treg, MAIT, and CTL.

The comparison of extrachromosomal circular DNA elements (eccDNA genes) found in the plasma of clinically diagnosed HUA patients with those in healthy individuals revealed exclusive expression of TLR6, IL2RA, PTGS1, MAPK13, and IL5 genes in HUA patients (95). Notably, IL5 and MAPK13 are integral to the operation of the IL-17 signaling pathway, which is closely associated with the T cell receptor signaling pathway. Consequently, These results suggest that eccDNA indicator assays related to T cells may have potential as novel organisms for early disease detection, risk assessment, and drug treatment response monitoring. However, because of the sample size of this study, further studies are needed for validation (95) (**Figure 3**).

## Imbalances in T cell populations play significant roles in various diseases

Alterations in T cell populations are observed in hyperuricemia and its related complications. The plasma of gout patients demonstrates enrichment of naїve CD4 T cells. In contrast, Th1/Th17 cells, effector memory CD8 T cells, and mucosal-associated invariant T cells (MAIT cells) are enriched in gout patients’ joint fluids (5). Recent studies have illustrated a close relationship between Th1/Th2 and Th17/Treg imbalance, both with hyperuricemia and its ensuing complications.

## Imbalance of Th1 and Th2

Excessive purines don’t directly enhance XOR expression in human hepatocytes; instead, they act on lymphocytes, escalating the production and release of IFN-γ. This highly expressed Interferon-gamma (IFN-γ) stimulates STAT1 and xanthine dehydrogenase(XDH) transcription in a manner dependent on IRF1, wherein STAT1 and IRF1 get attracted to the promoter, further recruiting CREB binding protein (CBP) to spur XDH transcription. This instigates XOR expression in hepatocytes, thus increasing UA production eventually (96). IFN-γ, majorly secreted by Th1 cells (97), implies that the activation of Th1 cells might be a potential pathogenic process, leading to HUA. In the case of HUA-afflicted mice, the spleen’s Th1 cells secrete inflammatory cytokines IL-1β and IFN-γ at higher concentrations, whereas Th2 cells secrete IL-4 and IL-10 at lower concentrations than the usual group. This finding indicates that modulating the Th1/Th2 equilibrium towards Th2 dominance can be a prospective strategy for HUA treatment (97). Yanjie et al. supported this in their study, using a novel multi-epitope vaccine to intensify the Th2 cytokines IL-10 and IL-4 in diabetic mice, amplifying the Th2-like immune response, and regulating the Th1/Th2 ratio imbalance. By making the immune response primarily Th2, it was possible to considerably lessen the UA levels in diabetic mice (98).

## Imbalance of Th17/Treg

Th17 and Treg represent two intimately related T cell variants. Individually, they perform integral functions in facilitating immune responses and repressing immunity. The coordination and balance between Th17 cells and Tregs enables the maintenance of standard immune functionality within the body (99). Microscopic crystalline MSU triggers an imbalance in the Th17/Treg ratio within human circulation, subsequently stimulating Th17 proliferation (45).

## Th17 cells

UA has been discovered to directly foster Th17 differentiation. A positive correlation has been established among the expression of lnc-NEAT1, HUA, Th17, and IL-17 (14). This correlation suggests that HUA may induce Th17 proliferation or activation through increased lnc-NEAT1 expression. It consequently yields an increased expression of the effector cytokine, IL-17, contributing to the development and onset of HUA conditions. Additionally, UA can indirectly instigate Th17 differentiation *via* other immune cells. CD4^+^ T cell polarization towards Th17 is triggered by MSU crystals, facilitated by DC cells (100). When co-cultured with DCs treated similarly, MSU crystals induce the latter to release IL-1α/β, prompting the activation of the NF-κB signaling pathway within CD4+ T cells. Consequently, this activation encourages the differentiation of CD4^+^ T cells into Th17 lineage and the secretion of the Th17-associated cytokine, IL-17A. The gut and its microbiota play a substantial role in HUA and its associated complications (101). Imbalance in gut tissue flora in HUA, leading to T-cell AKT/mTOR pathway activation, results in a higher number of Th17 and a decreased quantity of Treg, precipitating a Th17/Treg imbalance. Such an imbalance is marked by heightened expression of Th17 cytokines such as IFN-γ, TNF-α, IL-1β, IL-6, and IL-17. Consequently, this enhanced inflammatory response in the gut exacerbates the HUA conditions (101).

## Treg cells

Encouraging Treg differentiation presents a novel method for addressing HUA and its subsequent complications. The incidence of apoptosis, along with the depletion of circulating B-lymphocytes and the upregulation of Treg cells post-labrylase administration, proposes that this recombinant uricase treatment for HUA might influence outcomes by inhibiting B-cells to bolster Treg (102). These findings highlight the significance of manipulating the Th17/Treg balance as a treatment strategy for HUA.

## MAIT cells

MAIT cells are a unique subpopulation of innate invariant T cells, distinguished by the expression of an evolutionarily conserved invariant T cell receptor alpha (TCRα) chain. These cells interact with major histocompatibility complex class I-like molecules (MR1) and recognize ligands derived from bacteria or yeast, specific metabolites of riboflavin (vitamin B2) (103–105). Upon ligand recognition, cytokine signaling, or a combination of both, MAIT cells instantaneously manufacture Th1/Th17 cytokines, including IFN-γ, TNF-α, and IL-17, in an innate-like response. Young et al. discovered that the count of circulating MAIT cells in GA patients was remarkably lower than in a healthy population (16). However, GA patients displayed significantly elevated cell differentiation antigen CD69 expression levels, programmed cell death protein 1 (PD-1), and lymphocyte-activation gene 3 (LAG-3) in MAIT cells. Further studies demonstrated that MSU in the joint triggered the migration and activation of MAIT cells. Activated MAIT cells secrete pro-osteoclastogenic cytokines including IFN-γ, IL-6, IL-17, and TNF-α, which subsequently drive GA progression by promoting osteoclastogenesis and illustrating their movement from peripheral blood to inflamed areas. High levels of CC chemokine receptor 6 (CCR6) and C-X-C chemokine receptor type 6 (CXCR6) observed in circulating MAIT cells corresponded with the expression of their respective ligands CCL20 and CXCL16 around gouty tophi. Overall, MSU crystals instigate the migration of MAIT cells and stimulate their production of cytokines such as IFN-γ, TNF-α and IL-17, inducing T cell polarization towards pro-inflammatory Th1 and Th17 phenotypes. This mechanism contributes to the inflammatory responses observed in tissues.

## Cytotoxic T lymphocytes

An imbalance in CTLs, also contributes to T cells dysregulation. Frequently referred to as CD8^+^ T cells, CTLs are vital components of the adaptive immune system (106). Research has indicated that HUA disrupts the normal functions of CTLs and encourages their unchecked proliferation (107), thereby leading to dysregulated T cells proliferation.

## Innate lymphoid cells

### ILCs

ILCs, an integral part of tissue structures and resident cells, are lymphocytes that do not express many antigen receptor types found in T and B cells. These cells are pivotal in metabolism, tissue homeostasis, morphogenesis, and tissue repair and regeneration (108). An imbalance in the ILCs population and changes in its cytokine expression are observed in HUA and its complications. A subcategory of these, innate lymphoid cells type 3 (ILC3s), are a newly discovered group of innate immune cells implicated in the progression of multiple metabolic diseases. They achieve this through the secretion of IL-17 and IL-22. In HUA patients, an increased count of circulating ILC3s has been noted, correlating positively with serum UA and serum creatinine (Scr) levels. While there is no significant difference in plasma IL-17A concentrations between HUA patients and healthy individuals, a positive correlation exists between plasma IL-17A, serum UA concentrations, and circulating ILC3 frequencies in HUA patients. Therefore, ILC3s and IL-17A could serve as valuable indicators of disease severity and potential novel therapeutic targets for HUA. It has also been demonstrated that these ILC3 cytokines are associated with the severity and progression of HUA (109).

### Natural killer-cell

NK cells, a type of ILCs, play a vital role in immune surveillance as a component of the natural immune system (110). Typically, human NK cells are identified by the presence of CD56 proteins and the absence of CD3 proteins on their surface, and can be further subcategorized based on the relative expression of surface marker proteins CD56 and CD16, primarily into CD56 ^dim^ and CD56 ^bright^ subsets (111). NK cells are likely instrumental in the pathogenesis of HUA (112). For HUA patients with UA levels equal to or above 8.0 mg/dL, a decrease in the absolute number of CD3^-^ CD56^+^ NKG2D^+^ NK cells is observed, together with an increased quantity of CD107a-secreting NK cells (112). By impeding NK cell activation, it is feasible to augment Treg, thereby standardizing serum UA levels in uricase-deficient mice (113). This implies a correlation between the cell count and activation of NK cells and the development of HUA. Evidence suggests that NK cells observed at inflammation sites are initially recruited from the periphery, and, upon reaching, are further activated by site-specific cytokines. These activated NK cells establish interactions with other immune cells. Specifically, NK cells respond to a blend of IL-12 and IL-15 cytokines secreted by monocytes and macrophages by emitting IFN-γ, which can subsequently activate macrophages. This nexus between NK cells and macrophage and monocyte populations intensifies the production of inflammatory factors, thereby exacerbating the inflammatory response (114).

### UA activates the inflammatory response of tissue-intrinsic cells indirectly affecting immune cells

MSU crystals activate the TLR4/NF-κB/NLRP3 signaling pathway in human umbilical vein endothelial cells (HUVECs), leading to the secretion of intercellular cell adhesion molecule-1 (ICAM-1), IL-1β, IL-6, and vascular cellular adhesion molecule-1 (VCAM-1). The expression of these cytokines heightens the influx of neutrophils into the joint fluid, followed by an increased inflow of monocytes. When activated, monocytes and neutrophils actively engage in phagocytosis of MSU crystals, initiating an inflammatory response (115). Studies on HUA-induced cardiac remodeling have determined that sUA prompts cardiomyocytes to manufacture CXCL1 and CXCR2, which induce macrophage migration. This migration leads to an inflammatory response and results in cardiac myocyte hypertrophy (116).

In renal pathologies, sUA stimulates inherent renal cells, including endothelial cells, vascular smooth muscle cells, and renal tubular cells, to upregulate or secrete high mobility group protein 1 (HMGB1), inflammatory cytokines, and chemokines. These include C-reactive proteins, IL-6, IL-8, adhesion molecules such as ICAM-1 and VCAM-1, and monocyte chemotactic protein-1 (MCP-1). The expression of these molecules in endothelial cells facilitates macrophage alignment and immigration (117, 118). sUA also activates renal tubular TGF-β1/Smad, NF-κB, and Erk signaling pathways, prompting inflammatory cell secretion and subsequent macrophage migration (119–121). TLR4 receptor inhibition reduces MCP-1 secretion from sUA renal tubular cells (118). In addition, co-culturing macrophages with sUA-exposed renal tubular cells suggest that UA amplifies inflammatory responses by triggering renal lamina propria to produce pro-inflammatory factors, instigating M1 macrophages polarization (120). In the same vein, MSU crystals in renal disease incite an inflammatory response in renal parenchyma cells, promoting macrophages recruitment (122). This recruitment stems from MSU induced ICAM-1 expression in human mesangial cells, which fosters monocyte adhesion (117). Notably, gout studies disclose that MSU crystals activate fibroblast-like synoviocytes (FLS) JNK and ERK signaling pathways, augmenting neutrophil chemokine CXCL8 secretion. Consequently, elevated CXCL8 draws monocytes and neutrophils to the location of the urate crystals within bursal tissues, exacerbating inflammation (123).

Tamm-Horsfall protein (THP), alternatively known as uromodulin (UMOD), is a phosphatidylinositol-anchored glycoprotein exclusively formed by renal tubular cells (TAL) situated in the rising rough segment of the Henle loop (124). The function of UMOD as an immune activator has been authenticated (125), and UA linked injury to both TAL and its distal constituents precipitates increased interstitial UMOD. This prevalence, in turn, fosters the upregulation of pro-inflammatory cytokines such as TNF-α, IL-6, IL-8, and IL-1β (126). Moreover, UMOD overexpression correlates with diminished UA excretion, renal fibrosis, immune cell infiltration, and progressive renal failure. In the context of UA, THP adheres to monocytes, macrophages, and DCs, facilitating lymphocyte expansion (126) and mononuclear phagocytosis activation (127). Furthermore, THP within the renal mesenchyme enhancedmononuclear phagocyte quantity, plasticity, and phagocytic activity. An overnight incubation of THP with PBMCs promoted dose-dependent augmentations of IL-1β, IL-6, and TNF-α. Intriguingly, THP inhibits IL-1 initiated human T cell colony formation (124). Both UMOD and THP constitute pivotal proteins that modulate the impact of renal inherent cells on immune cells in hyperuric acid nephropathy, paving the way for insightful studies to clarify the origins of immune cell infiltration and uncover novel therapeutic strategies for HUA nephropathy.

The synovial fluid of HUA patients culled from joint tissues demonstrates an expression of RANTES and MCP-1, insinuating that UA may indirectly enhance the inflammatory response within the joint by elevating chemokine expression and enlisting monocytes. Accompanying studies examined in afflicted bursal tissues by gout underscore the function MSU crystals play in prompting FLS to produce cytokines IL-6, which further encourage Th17 differentiation and concurrently hinder the differentiation of regulatory T cells (123). Consequently, UA’s induction of immune cells through intrinsic cellular involvement presents a plausible mechanism through which UA contributes to GA (128).

## Summary

This critique examines the phenomena and chief mechanisms related to urate-induced dysregulation of immune cells. Distinct immune cells, including monocytes, macrophages, and T cells, extracted from patients suffering from HUA, are observed to undergo cellular activation and enhanced migratory effects, increased cytokine production, and problematic cellular differentiation when stimulated by urate *in vitro*. These instances of immune cell dysregulation are traced within the setting of MSU crystallization or sUA inflammatory models. Urate induced dysregulation of immune cells potentially spurs the onset of HUA and the consequent complications, besides bolstering chronic inflammation. Evidence of urate-induced long-term functional alterations also points to immune cell dysregulation, but further studies are needed to determine the metabolic adaptations of innate immune cells to urate as well as more in-depth studies to discuss the mechanisms by which UA regulates innate immunity. Morever, immune cells such as T cells and innate lymphocytes, which have only recently been discovered, still require a great deal of research to demonstrate the role and mechanisms of UA in their regulation. Our review indicates that consequent inflammation and tissue damage due to UA can be mitigated by suppressing specific immune cell populations or inhibiting the secretion of cytokines by immune cells.

Therefore, we may identify novel strategies for immunotherapeutic intervention in autoinflammatory diseases and their accompanying associated disorders by thoroughly understanding the mechanisms inherent to urate-induced dysregulation of immune cells and disease advancement.

## Author contributions

DeL: Conceptualization, Data curation, Formal Analysis, Writing – original draft. SY: Data curation, Formal Analysis, Writing – review & editing. YD: Formal Analysis, Supervision, Writing – review & editing. XC: Writing – review & editing. YL: Writing – review & editing. JO: Writing – review & editing. DaL: Writing – review & editing. HQ: Writing – review & editing. XF: Writing – review & editing. SW: Supervision, Writing – review & editing. XW: Supervision, Writing – review & editing. CL: Funding acquisition, Supervision, Writing – review & editing. WM: Funding acquisition, Supervision, Writing – review & editing.
